# Differences in Hydration Behavior and Physicochemical Properties of Starches from Different Sorghum Varieties and Their Multiple Relationships

**DOI:** 10.3390/foods14173131

**Published:** 2025-09-07

**Authors:** Jun Mao, Jihong Li, Hongdong Yan, Qun Shen

**Affiliations:** 1 College of Food Science and Nutritional Engineering, China Agricultural University, Beijing 100083, China; xjmaojun@hotmail.com; 2 Institute of Agricultural Products Processing, Xinjiang Academy of Agricultural Sciences, Urumqi 830091, China; 3 Jilin Academy of Agricultural Sciences, Gongzhuling 136100, China; nky106@163.com; 4 Institute of Crop Resources, Heilongjiang Academy of Agricultural Sciences, Harbin 150086, China; hljcrop@126.com

**Keywords:** wax sorghum starch, japonica sorghum starch, hydration behaviour, physicochemical properties, digestive properties

## Abstract

The physicochemical properties of sorghum determine its application. This study investigated the differences and relationships in the physicochemical properties of four typical japonica sorghum starches (JSS, including JZ159, JZ167, JZ169 and FZ4) and three waxy sorghum starches (WSS, including JN, LML and NL). The results showed that there were significant differences in their hydration properties, thermal properties, gelatinisation properties and digestibility. The water solubility index (WSI) of all starches was positively related to the swelling power (SP). With the increase in temperature (75–95 °C), the SP values of JSS and WSS samples increased significantly, and the SP of WSS samples was higher than that of JSS samples. Before 60 min, the digestibility of JZ159 and JZ167 was significantly lower. At 180 min of digestion, the digestibility of all starches was close (87.49–93.15%). At different temperatures, the SP and WSI values of WSS samples were significantly correlated with the predicted glycemic index (pGI), resistant starch (RS) and rapidly digestible starch (RDS), while the relationships among these parameters in JSS samples varied with temperature. This study also revealed the hydration characteristics of different sorghum starches and the influence of their hydration behaviours on physicochemical and digestive properties.

## 1. Introduction

Sorghum ranks fifth globally in terms of planting area and yield, following wheat, rice, corn, and barley [1]. In China, sorghum has a cultivation history of 5000 years. It is an important crop in the northeastern, northern, southwestern, and other regions of China, mainly distributed in provinces such as Inner Mongolia, Jilin, Guizhou, Heilongjiang, Liaoning, and Shanxi. Sorghum has garnered significant attention owing to its numerous health benefits [2]. As a gluten-free grain rich in resistant starch, sorghum contains abundant bioactive compounds that offer a wide range of physiological benefits, including anti-inflammatory, antioxidant, antithrombotic, and anti-diabetic properties, making it an important raw material source for functional foods [3].

Sorghum has a rich genetic diversity, with numerous varieties. Significant differences exist in the physicochemical properties of these varieties [4,5,6]. The primary component in sorghum grains is starch, with an average content of approximately 79.5% [7]. The physicochemical properties of starch were traditionally believed to be influenced by its amylose content (AC). However, the relationship between AC and the physicochemical properties of sorghum starch remains unclear [8]. Therefore, analyses of the physicochemical properties of starch in diverse sorghum varieties are of growing interest, as they can help breeders, food producers, and researchers make optimal choices when selecting varieties and designing products [5,9].

Hydration, including swelling and dissolution, is closely related to the processing quality and taste of starch-based foods and is a critical factor in many processes. Hydration can be used to predict the physicochemical and processing characteristics of starch [10,11]. The hydration of wheat has been used for rapid screening of wheat varieties [12]. Hydration is one of the most important structural characteristics of starch granules and is closely related to their chemical composition and physicochemical properties. Analyses of hydration can deepen the understanding of the characteristic relationships among the structural, physicochemical, and processing properties of starch granules [13].

Existing studies on the hydration behaviour of sorghum starch, particularly the relationship between its hydration behaviour and physicochemical properties, are scarce. However, the hydration behaviour of sorghum starch differs from those of wheat and maize starches [14], and hydration varies significantly among starch varieties [15]. For instance, although AC usually inhibits the swelling of starch [16], Singh et al. [17] observed no correlation between AC and swelling power (SP). Conversely, Boudries et al. [18] found that japonica sorghum starch with a higher AC had a higher SP. Grain starch usually shows a slow increase in SP at high temperatures (≥75 °C) [19], whereas waxy and japonica sorghum starch exhibit considerable increases at high temperatures [5,14,20]. Furthermore, the hydration behaviour of waxy starch, characterised by a high SP and low water solubility index (WSI), distinctly differs from that of high-amylose starch [21]. Nevertheless, existing studies on sorghum generally do not distinguish between starch types, which may lead to low or unestablished correlations between hydration and physiochemistry [5,17]. Therefore, studies should analyse the physicochemical properties of various types of sorghum starches and identify the relationship between their hydration, physicochemical, and digestive properties.

In summary, there is currently a lack of in-depth and detailed research on the hydration behavior of sorghum starch and its relationship with physicochemical properties, particularly regarding the differences in the correlation between hydration behavior and physicochemical characteristics across different temperatures and starch types. To address these issues, this study conducted a detailed analysis of the hydration behavior at various temperatures (75–95 °C) of seven typical sorghum starches, including three waxy sorghum starches (WSS; AC: 0–1.12%) and four japonica sorghum starches (JSS; AC: 24.89–29.67%), along with their physicochemical and digestive properties. Furthermore, the relationships between hydration behavior and physicochemical and digestive characteristics were systematically investigated, and differences between starch types were comprehensively evaluated.

## 2. Materials and Methods

### 2.1. Raw Materials

The raw materials were seven cultivated sorghum varieties, among which Jiza159 (JZ159), Jiza167 (JZ167), Jiza169 (JZ169), and Fengza4 (FZ4) were japonica cultivars; and Jinuo (JN), Longmiliang (LML), and Nuoliang (NL) were waxy cultivars. LML and NL were provided by Heilongjiang Academy of Agricultural Sciences, Harbin, China, whereas the other cultivars were provided by Jilin Academy of Agricultural Sciences. These varieties were planted from May to September 2023 in the Gongzhuling experimental field of the Jilin Academy of Agricultural Sciences (124°48′3″ E, 43°30′40″ N, altitude 224 m).

Amyloglucosidase (A7095, *Aspergillus niger*), *α*-amylase (A3172, porcine pancreas), pepsin (P7000), and invertase (I4504) were purchased from Sigma-Aldrich (St. Louis, MO, USA). Glucose content was determined using the glucose GOD-PAP assay kit (E1010, Beijing Prelein Gene Technology Co., LTD., Beijing, China). Other reagents were purchased from Beijing Chemicals Company (Beijing, China).

### 2.2. Starch Extraction and Analysis

The sorghum starch was extracted from sorghum grains using the alkaline steeping method described by Sun et al. [22]. The total starch content (TSC) of the extracted samples ranged from 94.88 to 97.84%. This high TSC indicates low levels of impurities, confirming that the extracted sorghum starch is suitable for further research. The starch characteristics of the sorghum varieties are presented in Table 1.

Total starch content (TSC) was determined enzymatically using the modified method presented by Goni et al. [23]. The AC was analysed using AACC method 61-03.01. Protein, crude fat, crude fibre, and ash content were analysed using AACC methods 61-03.01, 46-30.01, 30-25.01, 32-10.01, and 08-01.01, respectively. The amylopectin content (AP) is the difference between TSC and AC (TSC − AC). AC/AP is the ratio of AC to AP.

### 2.3. Morphological and X-Ray Diffraction Analysis

The granular morphology of starch was observed using a scanning electron microscope (SEM, Zeiss Merlin Compact, Jena, Germany) following the method described by Singh et al. [24]. The average particle size of starch was measured and calculated using Image J software v1.49 following the method reported by Yan et al. [5]. X-ray diffraction (XRD, Rigaku Company, Tokyo, Japan) was used to analyse the crystalline morphology and relative crystallinity (RC) of starch samples following the method presented by Singh et al. [24].

### 2.4. Analysis of Hydration Properties

The water absorption capacity (WAC) of starch was determined using an establish method [11]. 2 g of each starch (W) was mixed with 20 mL of distilled water to form a dispersion at room temperature, vortexed three times at low speed for 30 s, and allowed to stand for 10 min between each vortexing, followed by centrifugation at 3000× *g* for 30 min. The supernatant was discarded, and the weight of the precipitate (hydrated starch weight) (W_1_) was recorded, from which the mass of incorporated water (W_1_ − W) was calculated. The WAC value is expressed as g water/g starch. The following formula is used:

WAC = (W_1_ − W) ÷ W. (1)

The hydration properties of starch were characterised based on the SP, WSI, and close packing concentration (C*), which were determined and analysed according to the methods described by Tsai et al. [25], Mauro et al. [11], and Lowithun et al. [21], respectively. First, approximately 200 mg of the sample (W_0_) was mixed with 15 mL of distilled water in a 50 mL capped centrifuge tube to prepare a starch suspension. The suspension was incubated at 75 °C, 85 °C, and 95 °C for 30 min, with periodic inversion to maintain homogeneity. After incubation, the samples were cooled in an ice-water bath for 30 min and then centrifuged at 9000× *g* for 25 min. The supernatant was carefully aspirated, and the dissolved solids (W_1_) were obtained by drying to a constant weight at 105 °C. The weight of the centrifuged precipitate (W_2_) was recorded. The following formulas were used:

SP = W_2_ ÷ (W_0_ − W_1_), (2)

WSI = (W_1_ ÷ W_0_) × 100%, (3)

C* = (W_0_ ÷ W_2_) × 100%. (4)

### 2.5. Analysis of Functional Properties

Differential scanning calorimetry (DSC; Q2000, TA Instruments, New Castle, DE, USA) was used to analyse the thermodynamic properties of the samples according to the method presented by Gao et al. [26]. Specific method: Starch (3 mg) and distilled water (6 μL) were added to an aluminum sample pan, which was then hermetically sealed and equilibrated in a refrigerator at 4 °C for 24 h. The thermal scanning was performed in standard mode with a heating rate of 10 °C/min, ranging from 30 °C to 105 °C. Pasting properties were analysed using a Rapid Visco Analyzer (RVA; Model 4D, Newport Scientific, Liverpool, Australia) following the STD1 test detailed in AACC method 76-21.02. A portion of the RVA-gelatinised samples was subsequently used to measure the rheological properties with a dynamic rheometer (DHR-2, TA Instruments Ltd., Crawley, UK) equipped with a 40 mm parallel plate geometry and a 1 mm gap, following the method of Zhang and Shen [27]. Frequency sweep tests were performed within the linear viscoelastic region (the strain of 1%) over a frequency range of 0.1–10 Hz. Gels moulded into gel specimens measuring 20 mm (length) × 10 mm (width) × 10 mm (height) were stored at 4 °C for 24 h for gel stabilisation prior to analysis. Then, the texture properties of the gels were analysed using a texture analyser (TA.XT Plus, Stable Micro Systems, Godalming, UK) equipped with a P36/R probe. A two-cycle TPA was used, with the following test conditions: trigger force of 5 g, compression of 70% at rates of 1, 0.5, and 1 mm/s before, during, and after the test, respectively.

### 2.6. Analysis of Digestive Properties

Starch digestion fractions, including rapidly digestible starch (RDS), slowly digestible starch (SDS), and resistant starch (RS), were analysed using an established method [28]. The amount of starch sample used was 100 mg, and the total volume of the enzyme hydrolysis system was 25 mL, pH 5.2, containing *α*-amylase 189 U, invertase 37 U, and amyloglucosidase 2.6 U.

In vitro starch digestion was determined using a modified method [23]. The pepsin hydrolysis reaction had a total volume of 10 mL and contained 100 mg of starch sample and 20 mg of pepsin (250 U/mg) at pH 1.5. Samples were incubated at 40 °C for 1 h. Subsequently, *α*-amylase hydrolysis was carried out by adjusting the volume to 30 mL with a buffer solution (pH 6.9), adding α-amylase (2.6 U), and incubating at 37 °C for 3 h. Aliquots of 1 mL were withdrawn at 30, 60, 90, 120, and 180 min, then inactivated at 100 °C for 5 min. After cooling, 3 mL of buffer (pH 4.75) and 60 μL of amyloglucosidase (300 U/mL) were added, followed by incubation at 60 °C for 45 min. The hydrolysis index (HI) was calculated using the ratio of the area under the hydrolysis curve of starch to that of a reference sample (white bread). The predicted glycaemic index (pGI) was estimated based on the HI using the following formula:

pGI = 39.71 + 0.549 (HI).(5)

### 2.7. Statistical Analyses

Results were reported as means and standard deviations of at least three repeated measurements. An analysis of variance (ANOVA) using Duncan’s test (*p* < 0.05) was conducted in SPSS 23.0 (SPSS Inc., Chicago, IL, USA). Principal component analysis (PCA) and correlation analysis were performed using OriginPro 2024 following the method presented by Greenacre et al. [29]. The data were automatically normalized by the analysis software. Pearson correlation analysis was conducted, and correlation pie charts were generated using the CNSKnowAll platform (https://cnsknowall.com/#/Home/HighAll). By default, a network graph was used to display within-group correlation thresholds with an absolute value greater than 0.8 and *p* ≤ 0.05 (R ≥ 0.7).

## 3. Results

### 3.1. Starch Fractions

Table 1 shows the approximate composition of purified starch from seven tested sorghum varieties. TSC ranged from 94.88 to 97.84%, and no significant difference in TSC was observed among the seven sorghum varieties. The AC enabled clear classification of the tested varieties into three levels: FZ4 and JZ159 had the highest AC (28.46–29.67%), JZ167 and JZ169 had intermediate AC (24.89–24.96%), and waxy varieties (JN, LML, and NL) had minimal AC (0.27–1.12%).

### 3.2. Morphological and Structural Properties

**Figure 1:**
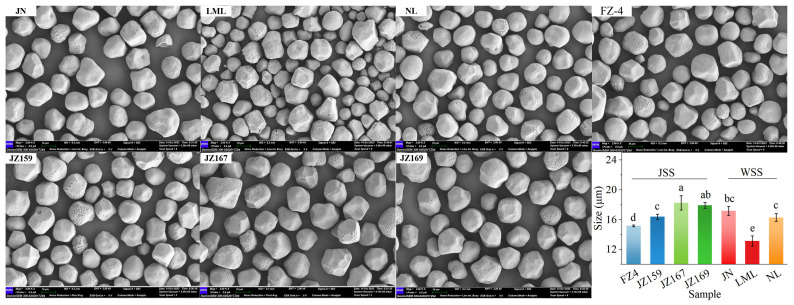
Scanning electron micrograph and size of starch granules from different sorghum cultivars (2000×). Different letters indicate significant differences (*p* < 0.05).

The SEM analysis revealed that the starch granules of the tested sorghum varieties predominantly exhibited irregular polygonal shapes with a minority being spherical. Notably, JZ167 and JZ169 displayed remarkably similar granule morphology. LML contained many small-sized granules, whereas JZ159 and FZ had fewer small granules than LML (Figure 1). The average granule sizes of WSS and JSS were 13.14–17.19 μm and 15.19–18.27 μm, respectively (Figure 1).

**Figure 2:**
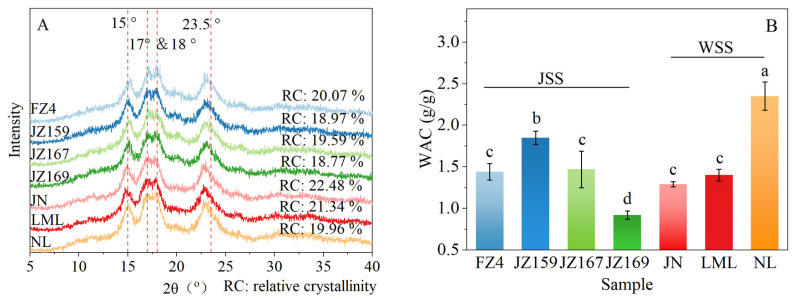
X-ray diffraction patterns (**A**) and WAC (**B**) of starches from different sorghum cultivars. Different letters indicate significant differences (*p* < 0.05).

The XRD patterns of all tested sorghum varieties exhibited four characteristic diffraction peaks at 15, 17, 18, and 23.5° (2θ). In addition, a weak dispersion peak was observed at 20° (2θ; Figure 2A). These similar diffraction features indicated that all examined starch samples possessed identical crystalline orientation. The RC of JSS and WSS were 18.77–20.07% and 19.9–22.48%, respectively (Figure 2A). FZ4 and JN had the highest crystallinity of JSS and WSS, respectively.

### 3.3. Hydration Properties

**Figure 3:**
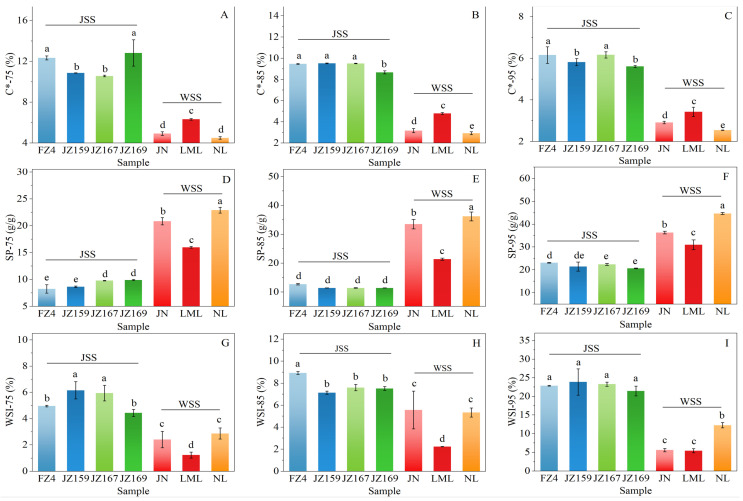
Variation trend of hydration parameters at different temperatures. (**A**–**C**): C*; (**D**–**F**): SP; (**G**–**I**): WSI. 75, 85, 95: Hydration temperature (°C). Different letters indicate significant differences (*p* < 0.05).

The WAC of WSS samples was 1.29–2.35 g/g, and JZ159 had the highest value (2.35 g/g; Figure 2B). The WAC of JSS samples was 0.92–1.85 g/g, and NL had the highest value (1.85 g/g). Figure 3 shows the variations in the C*, SP, and WSI values of the tested samples at various temperatures. Although overall C* decreased with increasing temperature, the C* trends of the JSS samples varied based on the temperature (Figure 3A–C). At 75–95 °C, C* was significantly lower in WSS than in JSS (*p* < 0.05). The C* of WSS samples remained consistent regardless of temperature.

The SP increased significantly at higher temperatures (Figure 3D–F). The SP of WSS samples was significantly higher than that of JSS samples (*p* < 0.05). For JSS, the increase in SP values was relatively modest between 75 and 85 °C but rose sharply from 85 to 95 °C. At 75 °C, JZ167 and JZ169 exhibited the highest SP values (9.78 and 9.90 g/g, respectively). In contrast, FZ4 showed the highest SP values at 85 °C (12.67 g/g) and 95 °C (23.12 g/g). The SP variation trend of WSS samples remained the same regardless of temperature. LML exhibited a similar SP variation pattern to that of the JSS samples. However, JN and NL exhibited the higher SP increases at 75–85 °C, with smaller increases at 85–95 °C. Thus, the starch varieties maintained a consistent order from highest to lowest SP values across all temperatures: NL > JN > LML.

The WSI values exhibited a similar variation trend to that of SP. The WSI values increased significantly at higher temperatures, and the discrepancy in the WSI values between JSS and WSS progressively increased with higher temperature (Figure 3G–I). The WSI values of JSS samples were significantly higher than those of WSS (*p* < 0.05; Figure 3G−I). The WSI values of JSS samples at 75, 85, and 95 °C were 4.44–6.16%, 7.14–8.93%, and 21.45–23.88%, respectively. The WSI values of JSS samples demonstrated an abrupt increase at 95 °C compared with those at 75 °C and 85 °C.

### 3.4. Functional Properties

#### 3.4.1. Thermal Properties

Table 2 summarised the starch gelatinisation characteristics, comprising onset temperature (To), peak temperature (Tp), conclusion temperature (Tc), gelatinisation enthalpy (ΔH), and gelatinisation temperature range (ΔT = Tc − To). Significant differences were observed in thermodynamic parameters among the seven sorghum varieties (*p* < 0.05). Except for ΔT, the WSS parameters (To, Tp, Tc, and △H) were significantly higher than those of JSS (*p* < 0.05). The ΔT values were 10.43–7.70 J/g in descending order: NL > FZ4 > LML > JZ169 > JZ159 > JZ167 > JN.

#### 3.4.2. Pasting Properties

**Figure 4:**
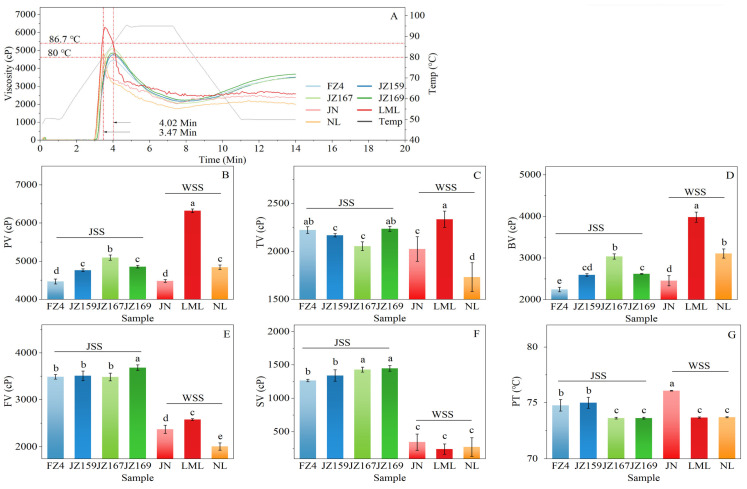
Pasting properties of starches from different sorghum cultivars: (**A**): pasting curves; (**B**): peak viscosity (PV); (**C**): trough viscosity (TV); (**D**): breakdown viscosity (BV); (**E**): final viscosity (FV); (**F**): Setback viscosity (SV); (**G**): pasting temperature (PT). Different letters indicate significant differences (*p* < 0.05). In Figure (**A**), 3.47 Min and 4.02 Min represent the shortest and longest time required for the test sample to generate PV, respectively; while 80 °C and 86.7 °C correspond to the lowest and highest temperatures at which PV formed, respectively.

The viscosity characteristics of the starch suspension varied significantly with temperature and time, as illustrated in Figure 4A. During the initial 10 min period, the pasting curves of JSS and WSS exhibited an intersecting pattern. Figure 4B–G show the variations in pasting parameters among samples. The peak viscosity (PV) values were 4469–6320 cP. LML had the highest PV value, whereas FZ4 exhibited the lowest value (4469 cP; Figure 4B). The final viscosity (FV) exhibited significant differences among sorghum starch varieties (Figure 4E). The FV values of JSS and WSS were 3485–3684 cP (JZ169 highest) and 2008–2580 cP (LML highest), respectively. FZ4 had the lowest pasting temperature (PT) value (74.77 °C) among JSS samples, whereas JN had the highest PT value (76.08 °C) among WSS samples (Figure 4G).

#### 3.4.3. Gel Texture Properties

The textural properties of the gels generated by RVA gelatinisation and cooling storage of the tested starches are presented in Table 3. Among WSS samples, the hardness, gumminess, and chewiness values of JN and LML were inferior. However, NL formed a softer paste than JN and LML, and its hardness, gumminess, and chewiness were below zero under the measurement conditions. Consequently, the gel properties could not be determined. The hardness, gumminess, and chewiness of JSS samples were significantly higher than those of WSS samples. However, the hardness, gumminess, and chewiness of JSS and WSS had similar variation trends.

#### 3.4.4. Rheological Properties

**Figure 5:**
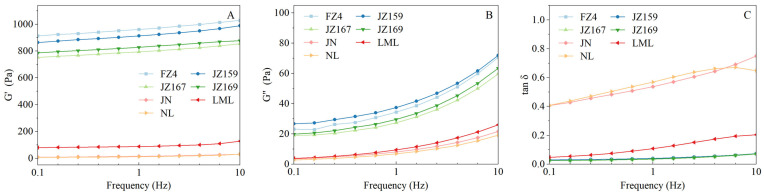
Rheological characterisation curves of starches from different sorghum cultivars. (**A**): the storage modulus (G′); (**B**): the loss modulus (G″); (**C**): the loss angle tangent (tan δ).

Figure 5A–C show the variation in storage modulus (G′), loss modulus (G″), and loss tangent (tan δ) of the samples, along with their frequencies. JSS samples had significantly higher G′ and G″ values than WSS samples (Figure 5A,B). The values of G′, G′′, and tan δ for all tested varieties at 1.0 Hz are presented in Table 4. The rheological characteristics of JSS samples differed from those of WSS samples. The G′ values of JSS samples were 793.81–961.29 Pa, with FZ4 exhibiting the highest value (961.29 Pa). The tan δ values of JSS samples were 0.03–0.04, showing no significant interspecific variation (*p* > 0.05). Conversely, the G′ and tan δ values of WSS samples were 12.12–87.97 Pa and 0.11–0.54, respectively. The tan δ values of WSS samples were significant (*p* < 0.05), whereas the G′ and G″ values were not (*p* > 0.05).

### 3.5. Digestive Properties

**Figure 6:**
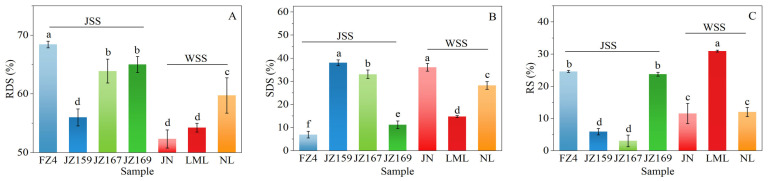
Starch digestion fractions of starch from different sorghum cultivars. (**A**): Rapidly digestible starch (RDS); (**B**): slowly digestible starch (SDS); (**C**): resistant starch (RS). Different letters indicate significant differences (*p* < 0.05).

The digested fractions of the samples’ starch, comprising RDS, SDS, and RS, are shown in Figure 6. The RDS, SDS, and RS values differed significantly between JSS and WSS (*p* < 0.05). The RDS, SDS, and RS values of JSS samples were 55.99–68.42% (highest in FZ4), 6.96–38.07% (highest in JZ159), and 3.06–24.62% (highest in FZ4), respectively. Conversely, the RDS, SDS, and RS values of WSS were 52.32–59.74% (highest in FZ4), 14.80–36.11% (highest in NL), and 11.56–30.95% (highest in JN), respectively. The variations in SDS and RS values of WSS and JSS samples showed opposite trends (Figure 6B,C).

**Figure 7:**
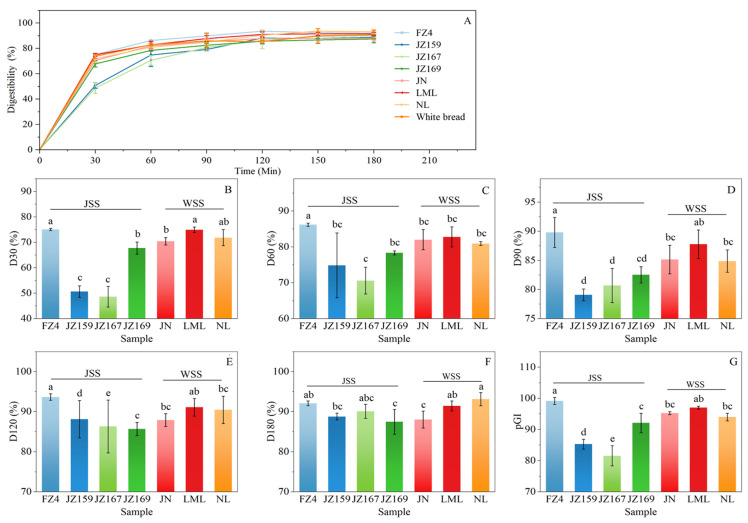
In vitro digestion characteristics of starch from different sorghum cultivars. (**A**): digestion curve; (**B**–**F**): digestibility from 30 to 180 min; (**G**): pGI. Different letters indicate significant differences (*p* < 0.05).

Figure 7A shows the time-dependent hydrolysis profiles of the tested starches over 180 min. All starches showed a progressive increase in digestibility during the initial 120 min, followed by stabilisation, with final digestibility converging to similar levels (87.49–93.15%). Figure 7B–F show the variation in digestibility at each time point for each variety. In the first 120 min, FZ4 exhibited the highest digestibility at 30 (79.09%), 60 (86.18%), 90 (89.79%), and 120 (93.67%) min. Prior to 90 min, the digestibility of JZ159 and JZ167 was lower than that of other varieties, especially at 30 (50.65%, 48.63%) and 60 (74.84%, 70.61%) minutes. In addition, FZ4 had the highest pGI value (99.16), whereas JZ159 and JZ167 had the lower pGI values (85.31 and 81.57), which were consistent with the digestibility analysis results (Figure 7G).

### 3.6. PCA and Correlation Analysis

**Figure 8:**
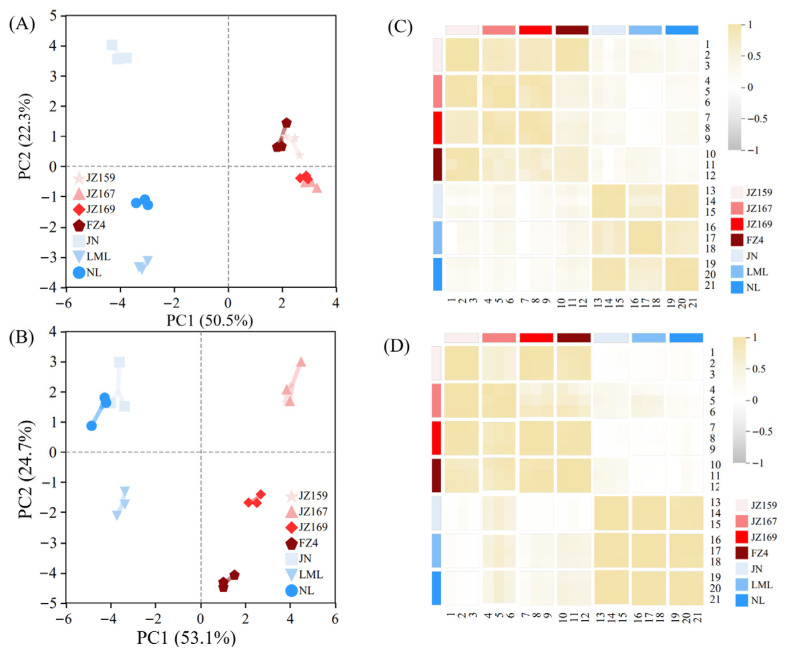
Results of Principal Component Analysis. (**A**,**B**) Composed of chemical composition and particle size from Table 1 and Figure 1, thermal properties from Table 2, and hydration properties from Figure 3. (**C**,**D**) Composed of pasting properties and digestion properties from Figure 4, Figure 6 and Figure 7.

The PCA of starch composition, grain size, and thermal and hydration properties revealed that two principal components (PC1 and PC2) explained 72.8% of the total variance in the seven varieties of sorghum samples (Figure 8A). The PCA of pasting and digested fractions explained 78.8% of the total variation among all the tested varieties (Figure 8C). These results identified the effective discrimination of the seven sorghum samples. JSS samples were mainly distributed in the first and fourth quadrants, whereas WSS samples were mainly distributed in the second and third quadrants. Based on the related properties, the seven varieties of sorghum samples were clustered in R-type using a systematic cluster analysis. The clustering method was inter-group connection, and the clustering interval was Pearson correlation. The seven sorghum samples were divided into two clusters. The significant divergence of samples into clusters verified the PCA results (Figure 8B,D).

**Figure 9:**
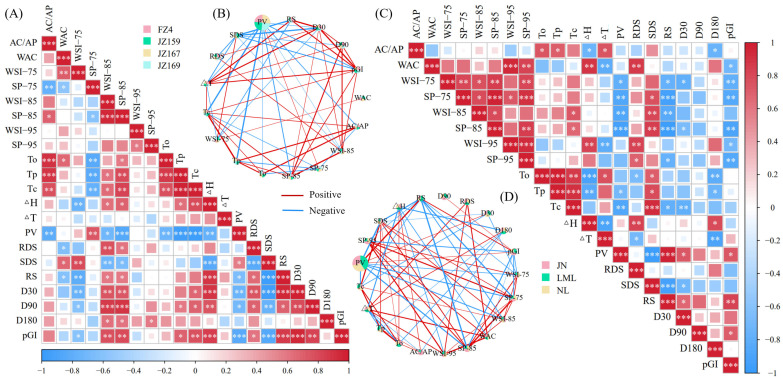
Correlation analysis between physicochemical properties. (**A**) JSS; (**B**) the correlation pie chart of JSS; (**C**) WSS; (**D**) the correlation pie chart of WSS. * *p* ≤ 0.05, ** *p* ≤ 0.01, *** *p* ≤ 0.001.

A universally positive correlation was observed between the hydration characteristics of WSS samples regardless of temperature. In contrast, a negative correlation was observed between the hydration characteristics of JSS samples (Figure 9A,B). The SP values of JSS were significantly and negatively correlated with AC/AP at 75 °C, and significantly and positively correlated with it at 85 °C (*p* < 0.05). The hydration characteristics of WSS were consistent with thermal properties, PV, and digestion fraction correlation (positive or negative), regardless of temperature. However, the situation differed for JSS. The SP value was significantly negatively correlated with PV at 75 °C (*p* < 0.05), but significantly positively correlated with it at 85 °C (*p* < 0.05). Among all the samples, AC/AP was significantly correlated with To, Tp and Tc, indicating that amylose has a significant impact on the thermal properties of starch granules, even in WSS samples with very low AC. Only Tc was significantly positively correlated with SP and WSI of 85 °C in WSS (*p* < 0.05), but To, Tp, and Tc were negatively and positively correlated with SP at 75 °C and SP and WSI at 85 °C in JSS (*p* < 0.05), respectively. The association between hydration properties and digestive characteristics of WSS and JSS differed. The correlations between SP, WSI and RDS, SDS, and RS in WSS were consistent across different temperatures. Increases in SP and WSI led to elevated RDS and SDS, but a reduction in RS. For JSS, however, RDS and RS showed negative correlations with SP of 75 °C, but positive correlations with both SP and WSI at 85 °C. In contrast, the trend for SDS was the opposite. The pGI values were negatively correlated with hydration characteristics in WSS samples, whereas the pGI values of the JSS samples were negatively and positively correlated with the hydration characteristics at 75 and 85 °C, respectively.

The correlation heat map results were screened. The PCA and correlation analysis results were supported by a network correlation (R ≥ 0.8, *p* < 0.05). In all samples, PV had the largest weight in the pie chart of network relevance and the strongest correlation with hydration characteristics (SP and WSI) at 85 °C, indicating that the hydration characteristics at this temperature have the strongest correlation with other physicochemical properties. This was consistent with the correlation heat map results (Figure 9B,D).

## 4. Discussion

### 4.1. Morphological and Structural Properties of Sorghum Varieties

Different crystal types may lead to variations in hydration capacity and behaviour [13]. Starch crystallinity is generally classified into three types: A-type, B-type, and C-type. A-type starch exists mainly in cereal crops [30]. All samples showed characteristic XRD diffraction peaks at 15, 17, 18, and 23.5° (2θ), consistent with the findings of Singh et al. [24] for sorghum starch. This indicates that all samples shared the same crystalline structure and were classified as A-type starch. All samples had similar morphology of starch granules but different sizes, as they were isolated from the same plant. Some studies have suggested that the RC and size of starch particles are affected by the AC [31]. However, no corresponding relationship was found among the samples. Kang et al. [32] noted that the particle size and RC of sorghum starch are determined by multiple factors, such as genotype, cultivation methods, grain maturity period, and molecular structure. Furthermore, some studies have shown that RC and particle size affect the hydration, thermodynamics, gelatinisation, digestive, and other properties of starch [5,33].

### 4.2. Hydration Behaviours of Sorghum Varieties

The SP of starch granules is mainly determined by their amylopectin content, with amylose having an inhibitory effect [34]. Moreover, the SP is affected by the AC/AP [19]. However, at high temperatures (≥85 °C), waxy starch may present SP equivalent to that of high amylose [11,13]. The SP values of WSS samples were significantly higher (*p* < 0.05) than those of JSS samples (*p* < 0.05) at 75–95 °C, which was similar to the observation on both types of sorghum starch by Yan et al. [5]. The WSI values of WSS samples were significantly lower than those of JSS samples, likely because JSS samples were rich in amylose. The leaching of amylose leads to an increase in the WSI values of samples with a higher AC [35].

Hydration characteristics are affected by temperature, increasing at higher temperatures [36]. The hydration characteristics of most cereal starches increase significantly at 50–60 °C [19]. All samples showed a significant increase in SP values at 75–95 °C. In particular, the JSS samples showed a dramatic increase (approximately two-fold) at temperatures above 85 °C. Previous studies have reported similar results for sorghum [5] and rice starch [37,38]. This is suggested to be related to the internal structure of the starch granules [14] and their molecular fine structure [37,38]. The significant increase in the AC/AP resulted in significantly lower SP values in JSS samples than in WSS samples. However, FZ4 and JZ159 in samples with higher AC/AP had higher SP values at high temperatures (≥85 °C), which was in line with the findings of Carcea et al. [14] and Boudries et al. [18] for JSS. Research on rice has suggested that starch swelling is affected by varying factors during heating: at low temperatures (50–70 °C), starch swelling is influenced by the short chains of amylopectin, whereas at high temperatures (80–90 °C), it is affected by AC [37,38]. The results indicated that the relationship between the SP and AC/AP of JSS samples was temperature-dependent. The SP of WSS samples maintained a similar trend at all temperatures. The SP values of LML were significantly lower than those of JN and NL, indicating significant differences in their amylopectin structure, as they contained no or very little amylose. It was concluded that SP was positively correlated with the short branch chains of amylopectin and negatively correlated with the long and extra-long chains [33,34].

The WSI and AC/AP trends were not synchronous between varieties, suggesting a lack of a significant correlation between them. This was in line with the findings regarding sorghum starch presented by Yan et al. [5]. Studies have shown that the WSI is affected by intermolecular forces [39], and the SP is positively correlated with the WSI [40]. The present study found that the WSI and SP values of WSS samples exhibited similar variations; however, similarities were observed for JSS only at 85 and 95 °C. This aligned with the findings of Carcea et al. [14].

### 4.3. Functional Properties of Sorghum Varieties

Studies have suggested that gelatinisation leads to elevated SP and WSI [41]. Among WSS samples, NL had the lowest To and ΔH values and the highest hydration capacity (based on SP and WSI parameters) at 75, 85, and 95 °C. Among JSS samples, FZ4 and JZ169 exhibited a similar phenomenon at 75 °C. In contrast, FZ4 and JZ169 showed the opposite trends at 85 °C. Specifically, FZ4 had the highest Tp and Tc values, and its SP and WSI values were the highest at 85 °C. JZ167 had the lowest Tp and Tc values, and its SP value was the lowest at 85 °C. These results indicated that the hydration properties of starch granules were related to their structure and organisation. Gelatinisation and hydration are essentially reactions of the interactions between starch and water molecules and between amorphous and crystalline regions [19,42]. Gelatinisation and hydration are influenced by factors such as the molecular structure of amylopectin, AC/AP, and the size of starch granules [19]. Among JSS samples, FZ4 showed a distinct correspondence between hydration characteristics at various temperatures and the gelatinisation temperature. This may be influenced by the differences in hydration behaviours at various temperatures, as starch gelatinisation may be mainly driven by hydration [19].

Starch viscosity increases gradually with increasing suspension temperature and expanding starch particles. PV values were obtained when the maximum swelling potential was reached. Subsequently, some granules begin to break up and lose their rigid structure, the starch paste forms a uniform mass, and viscosity decreases [13]. SP values are generally positively correlated with PV values [11]. In the present study, although LML had the highest PV value among all samples, the PV values of JN and NL were significantly lower than those of JZ167 (*p* < 0.05). This was in line with previous findings for sorghum [17] and rice [43]. This is related to the differences in the dominant structural features under different starch concentrations [44]. Viscosity, which is a rheological property, is affected by starch concentration (C): when C < C*, PV is determined by volume fraction, whereas when C > C*, PV is determined by granule rigidity [21]. At the temperature at which PV occurred, C* values were lower than C for all samples (10.7%), indicating that PV was mainly affected by particle rigidity. The SP is generally negatively correlated with rigidity [19]. For WSS, PV and SP values showed a clear inverse trend. However, for JSS samples, JZ167 and JZ169 had the higher PV values, whereas their SP values at 75 and 85 °C were the highest and lowest, respectively. Similar results have been reported for rice starch [37,38]. The smaller difference between C* and C of JSS than of WSS may have caused the relationship between the SP and PV relationship of JSS samples to be affected by the hydration temperature.

The gel and rheological properties of starch are correlated with the volume fraction and rigidity of swollen particles [13,17]. In WSS samples, the variations in hardness among samples were inversely proportional to the SP values. Therefore, the gel of NL being too soft can be attributed to its high SP, similar to the waxy rice starch samples measured by Wang et al. [37]. In contrast, the variations in hardness among JSS samples were inversely proportional to SP values only at 75 °C. This may be related to the differences in the hydration behaviours of JSS samples based on temperature. The G′ and G″ of all samples increased with the increase in frequency, showing frequency dependence. The G′ value of JSS was much higher than that of WSS, whereas Tan δ showed the opposite trend. This suggests that JSS formed a firm gel, while WSS formed only a soft paste. In WSS samples, the trends of G′ and G″ were inversely related to SP values, whereas Tan δ displayed the opposite. Compared to JN and NL, LML had significantly increased G′ value and decreased Tan δ value, indicating that it may have more long-chain structure because they have the same AC. The long chains of amylopectin can reduce starch swelling by entanglement [16,21]. In contrast, the overall trends for G′ and G″ for JSS were only inversely proportional to the SP value at 75 °C. There was no significant difference in Tan δ values between samples.

### 4.4. Digestive Properties of Sorghum Varieties

Amylose is difficult to digest owing to its strong molecular structure, leading to a slow digestion rate [45]. In this study, the RDS content of the JSS samples was the highest overall among the samples. This aligned with the findings for WSS and JSS presented by Yang et al. [8]. Z159 and JZ167 had the lowest RS content, whereas LML had the highest RS, which was in line with the observation on rice starch reported by Lin et al. [43]. Low swelling may lead to difficulty in diffusion of enzymes into starch granules, resulting in lower hydrolysis rates [46]. According to Sharma et al. [47], the digestion rate is mainly associated with WSI. Among WSS samples, the WSI and RDS values of NL were the highest, whereas its RS value was the lowest. Furthermore, the SP and WSI values of LML were lowest, whereas its RS content was the highest. Among JSS samples, FZ4 had the highest SP and WSI at high temperatures (≥85 °C), and its RDS was the highest. JZ159 and JZ167 had the highest WSI at low temperatures (75 °C), and their RS content was the lowest.

At 180 min, the total digestibility was similar between all samples, which aligned with the findings of Kang et al. [32]. Among WSS samples, LML had the highest digestibility and pGI, whereas its SP and WSI values were the lowest. Studies have shown that increased digestibility of WSS is associated with an increase in long side chains of amylopectin [32]. However, an increase in the long side chains of amylopectin also leads to a decrease in the SP [16,34]. Among JSS samples, the digestibility and pGI of FZ4 were the highest, which could be attributed to their highest SP and WSI at high temperature (≥85 °C) [46].

### 4.5. Correlations Between Physicochemical Characteristics

PCA and cluster analysis were used to distinguish the seven sorghum samples. JSS and WSS exhibited the highest similarity within their respective types. The AC/AP was significantly correlated with several important parameters. Specifically, the AC/AP was positively correlated with gelatinisation temperature in all samples, indicating that the presence of AC improved the thermal stability of sorghum starch. This may be related to the entanglement of amylose or formation of a partial helix between amylose and amylopectin in amorphous sheets to strengthen the particle structure [34]. In JSS samples, the AC/AP was negatively correlated with PV (*p* < 0.05). This may be because amylose enhances the thermal stability of starch granules and inhibits the expansion of amylopectin, thereby decreasing viscosity. In contrast, in WSS samples, the AC/AP was not correlated with PV. This may be because the AC in WSS is not sufficient to significantly affect the swelling of starch granules [48,49].

In JSS samples, the AC/AP was negatively correlated with the SP at 75 °C, positively correlated with the SP at 85 °C (*p* < 0.05), and not correlated with the SP at 95 °C. This was likely because at higher temperatures, the expansion destroyed the stability of amylopectin microcrystals. At 75 °C, the expansion destroys the small microcrystals. At 85 °C, larger microcrystals are destroyed. Amylose mainly exists in larger microcrystals, resulting in amylose leaching at high temperatures (≥85 °C). This aligns with the findings of Carcea et al. [14] and Ratnayake [35]. The fluctuations in the correlation between the AC/AP and SP at various temperatures revealed the crystal composition and distribution of amylose. In contrast, no correlation was observed between the AC/AP and SP in WSS samples. Their hydration behaviours were likely influenced by the fine structure of amylopectin [49,50] rather than being related to their content.

Hydration characteristics determine the function, nutrition, and processing properties of starch, which are closely associated with the starch structure [42]. In JSS samples, the SP was positively correlated with gelatinisation temperature and negatively correlated with PV at a hydration measurement temperature of 75 °C (*p* < 0.05). At 85 °C, the SP and WSI were negatively correlated with PV (*p* < 0.05), whereas this correlation was not significant at 95 °C. In WSS samples, the SP and WSI were not significantly correlated with the gelatinisation temperature. This was likely because the high expansion and dispersion ability of waxy starch reduced the influence of their structural characteristics. Moreover, at 75 and 85 °C, the WSI and SP of WSS varieties were significantly and negatively correlated with PV. This correlation weakened at 95 °C, likely due to the high expansion of waxy starch, which led to PV associated with particle rigidity [10].

The hydration and digestive characteristics of starch are interrelated [13,46]. The SP and WSI of JSS samples were positively correlated with pGI only at 85 °C, whereas the SP and WSI of WSS samples were negatively correlated with pGI at various hydration temperatures (*p* < 0.05). Previous studies have shown that increased digestibility of WSS is associated with the increase in long side chains of amylopectin [32]; however, this increase also decreases the SP [50,51].

Hydration properties can also serve as indicators of starch application potential. The low SP and high WSI values of JSS samples suggest they are better suited for products requiring low viscosity and firm texture, and they may also contribute to sweetness [52]. In contrast, WSS samples have opposite properties, indicating a greater suitability for high-viscosity applications [53]. Furthermore, the high RS of LML confers potential application value in developing healthy foods with high viscosity. This is attributed to the remarkable physiological benefits of resistant starch, including enhanced intestinal barrier function, reduced inflammation and obesity, and improved insulin resistance [54].

## 5. Conclusions

The morphology and physicochemical properties of seven sorghum starches were systematically analysed. The results demonstrated that the starch granules of sorghum varieties showed the characteristics of an A-type starch, with similar irregular morphology and varying sizes. The starches isolated from the sorghum varieties exhibited different thermal behaviours during gelatinisation. The thermal properties indicated that the starch isolated from JN, a WSS sample, exhibited the highest stability under heating. At 75–97 °C, the hydration of WSS differed from that of JSS, and WSS has a high hydration ability. The hydration characteristics indicate that the NL variety is a potential candidate raw material for producing high-viscosity foods. Meanwhile, the higher resistant starch content in LML suggests its potential application value in developing functional foods for regulating dietary health. The relationship between the hydration and physicochemical characteristics of WSS was consistent regardless of temperature. Conversely, the relationship between the hydration and physicochemical characteristics of JSS differed based on temperature. In addition, a strong correlation was observed between the AC/AP and several functional characteristics of starch. Thus, the AC/AP could be used as an index of starch application in industry.

## Figures and Tables

**Table 1:** Approximate starch fraction of different sorghum varieties.

Type	Sample	TSC	AC	AC/AP	Protein	Fiber
JSS	FZ4	94.88 ± 0.99a	29.67 ± 0.94a	0.46 ± 0.02a	0.12 ± 0.01d	0.17 ± 0.01a
	JZ159	96.03 ± 0.17a	28.46 ± 1.36a	0.42 ± 0.03a	0.15 ± 0.01c	0.15 ± 0.01b
	JZ167	96.58 ± 1.00a	24.96 ± 1.76b	0.35 ± 0.03b	0.16 ± 0.01b	0.15 ± 0.01b
	JZ169	97.84 ± 1.72a	24.89 ± 1.76b	0.34 ± 0.02b	0.17 ± 0.01b	0.15 ± 0.01b
WSS	JN	96.08 ± 1.95a	1.12 ± 0.79c	0.01 ± 0.01c	0.22 ± 0.01a	0.17 ± 0.01a
	LML	96.97 ± 0.92a	0.28 ± 0.48c	0.003 ± 0.01c	0.06 ± 0.01f	0.17 ± 0.01a
	NL	95.78 ± 2.78a	0.27 ± 0.23c	0.003 ± 0.00c	0.09 ± 0.01e	0.16 ± 0.01a

Analysed using one-way ANOVA Duncan Multiple Range Test, different letters in the same column indicate significant differences (*p* < 0.05, *n* = 3). JSS: high-amylose sorghum cultivars; WSS: Waxy sorghum cultivars; TSC: Total starch content; AC: amylose content; AC/AP = AC/(TSC − AC).

**Table 2:** Thermal properties (DSC) of starches from different sorghum cultivars.

Type	Sample	To (°C)	Tp (°C)	Tc (°C)	∆H (J/g)	△T (°C)
JSS	FZ4	68.33 ± 0.21d	72.83 ± 0.25c	77.77 ± 0.46d	12.92 ± 0.07de	9.43 ± 0.45b
	JZ159	68.30 ± 0.10d	72.10 ± 0.00d	76.53 ± 0.21e	13.21 ± 0.04cd	8.23 ± 0.25c
	JZ167	67.17 ± 0.25e	70.63 ± 0.21f	74.87 ± 0.21g	12.48 ± 0.36ce	7.70 ± 0.26d
	JZ169	66.83 ± 0.06f	71.13 ± 0.06e	75.83 ± 0.25f	13.18 ± 0.93d	9.00 ± 0.20b
WSS	JN	73.13 ± 0.15a	76.03 ± 0.15a	80.87 ± 0.12a	15.62 ± 0.40a	7.73 ± 0.06d
	LML	69.53 ± 0.21b	73.23 ± 0.31b	78.67 ± 0.35c	14.08 ± 0.44b	9.13 ± 0.23b
	NL	69.13 ± 0.12c	73.40 ± 0.10b	79.57 ± 0.25b	13.61 ± 0.23bc	10.43 ± 0.15a

Analysed using one-way ANOVA Duncan Multiple Range Test, different letters in the same column indicate significant differences (*p* < 0.05, *n* = 3). To: onset temperature; Tp: peak temperature; Tc: conclusion temperature; ∆H: gelatinisation enthalpy; △T = Tc − To.

**Table 3:** Textural properties of starch gels from different sorghum cultivars.

Type	Sample	Hardness (N)	Gumminess (N)	Chewiness (N)	Springiness	Cohesiveness	Resilience
JSS	FZ4	10.92 ± 0.11a	8.87 ± 0.29a	8.16 ± 0.31a	0.92 ± 0.01b	0.81 ± 0.02b	0.54 ± 0.01c
	JZ159	10.56 ± 0.59ab	8.75 ± 0.32a	8.24 ± 0.21a	0.94 ± 0.02b	0.83 ± 0.02b	0.59 ± 0.02b
	JZ167	10.02 ± 0.66bc	8.23 ± 0.38b	7.70 ± 0.32b	0.94 ± 0.01b	0.82 ± 0.02b	0.56 ± 0.01bc
	JZ169	9.64 ± 0.46c	8.03 ± 0.38b	7.54 ± 0.38b	0.94 ± 0.01b	0.84 ± 0.01b	0.57 ± 0.03bc
WSS	JN	0.72 ± 0.13d	0.64 ± 0.10c	0.71 ± 0.1c	1.11 ± 0.21a	0.89 ± 0.04a	0.59 ± 0.03b
	LML	1.31 ± 0.10d	1.07 ± 0.12c	1.02 ± 0.10c	0.95 ± 0.05b	0.83 ± 0.01b	0.67 ± 0.04a
	NL	/	/	/	/	/	/

Analysed using one-way ANOVA Duncan Multiple Range Test, different letters in the same column indicate significant differences (*p* < 0.05, *n* = 3). “/”: Undetermined.

**Table 4:** Rheological characteristics of starch gels from different sorghum cultivars.

Type	Sample	G′ (Pa)	G″ (Pa)	Tan δ
JSS	FZ4	961.29 ± 50.63a	34.36 ± 2.54ab	0.04 ± 0.01d
	JZ159	912.24 ± 110.58ab	37.45 ± 7.96a	0.04 ± 0.00d
	JZ167	793.81 ± 58.98c	27.43 ± 1.54c	0.03 ± 0.01d
	JZ169	828.33 ± 8.42bc	29.58 ± 0.91bc	0.04 ± 0.00d
WSS	JN	15.12 ± 0.15d	8.12 ± 0.15d	0.54 ± 0.01b
	LML	87.94 ± 10.19d	9.41 ± 0.72d	0.11 ± 0.01c
	NL	12.12 ± 0.29d	6.89 ± 0.10d	0.57 ± 0.01a

Analysed using one-way ANOVA Duncan Multiple Range Test, different letters in the same column indicate significant differences (*p* < 0.05, *n* = 3). G′: storage modulus; G′′: loss modulus; tan δ: loss angle tangent.

## Data Availability

Data is contained within the article.

## Abbreviations

The following abbreviations are used in this manuscript:

AC	Amylose content
SP	Swelling power
WSI	Water solubility index
JSS	Japonica sorghum starches
WSS	Waxy sorghum starches
JZ159	Jiza159
JZ167	Jiza167
JZ169	Jiza169
FZ4	Fengza4
JN	Jinuo
LML	Longmiliang
NL	Nuoliang
A7095	Amyloglucosidase
A3172	*α*-amylase
P7000	Pepsin
P7545	Pancreatin enzyme
TPC	Total polyphenol content
TC	Tannins content
TSC	Total starch content
SEM	Scanning electron microscope
XRD	X-ray diffraction
RC	Relative crystallinity
WAC	Water absorption capacity
C*	Close packing concentration
DSC	Differential scanning calorimetry
RVA	Rapid Visco Analyzer
RDS	Rapidly digestible starch
SDS	Slowly digestible starch
RS	Resistant starch
HI	Hydrolysis index
pGI	Predicted glycaemic index
ANOVA	Analysis of variance
PCA	Principal component analysis
To	Onset temperature
Tp	Peak temperature
Tc	Conclusion temperature
ΔH	Gelatinisation enthalpy
ΔT	Gelatinisation temperature range
G′	Storage modulus
G″	Loss modulus
tan δ	Loss tangent
AC/AP	Ratio of amylose to amylopectin
C	Starch concentration
